# Enhanced efficacy of *Magnolia denudata* essential oil in fish anesthesia using nanoemulsions and self-microemulsifying drug delivery systems

**DOI:** 10.3389/fvets.2024.1440275

**Published:** 2024-11-27

**Authors:** Xiangbing Zeng, Hongbiao Dong, Xiaoting Zheng, Jiasong Zhang

**Affiliations:** ^1^Key Laboratory of South China Sea Fishery Resources Exploitation and Utilization, Ministry of Agriculture and Rural Affairs, South China Sea Fisheries Research Institute, Chinese Academy of Fishery Sciences, Guangzhou, China; ^2^Key Laboratory of Mariculture, Ministry of Education, Ocean University of China, Qingdao, China; ^3^Key Laboratory of Efficient Utilization and Processing of Marine Fishery Resources of Hainan Province, Sanya, China

**Keywords:** anesthetic, essential oil, fish, nanoemulsion, physiological effect

## Abstract

**Introduction:**

The use of plant essential oils as anesthetics for fish has gained increasing attention, but ethanol, often used as a co-solvent, presents certain limitations. Recently, *Magnolia denudata* essential oil (MDO) has emerged as a promising alternative for fish anesthesia and sedation.

**Methods and results:**

To further improve MDO anesthesia efficacy, this study developed nanoemulsion (NE) and self-microemulsifying drug delivery system (SMEDDS) formulations of MDO. Transmission electron microscopy and stability tests confirmed that both NE and SMEDDS possess smaller particle sizes and are stable under various temperature conditions. Anesthetic trials on fish demonstrated that these formulations reduced the time needed to induce anesthesia compared with the non-formulations. Additionally, physiological assessments of the fish gills showed that neither NE nor SMEDDS caused irreversible damage to respiratory function.

**Discussion:**

Overall, NE and SMEDDS present a safe and effective delivery system for MDO, enhancing its anesthetic properties while minimizing potential harm to aquatic organisms compared to traditional methods.

## 1 Introduction

Essential oils are derived from the leaves, flowers, buds, and stems of plants, with some possessing anesthetic properties for fish. In addition to their anesthetic effects, these oils offer antibacterial, antioxidant benefits and pose minimal health risks to humans (1–4). However, their poor water solubility and high volatility limit their application in aquaculture (1, 5, 6). To be used in aquatic environments, essential oils must first be dissolved in water-soluble organic solvents. Unfortunately, high concentrations of solvents like ethanol can induce excessive excitement and activity in fish (7). This challenge can be addressed by formulating essential oils into delivery systems with minimal or no organic solvents, such as nanoemulsions (NE) or self-microemulsifying drug delivery systems (SMEDDS) (7, 8).

Nanoemulsions are emulsions with very small droplet sizes, typically between 10 to 150 nm. These systems, also known as microemulsions, ultrafine emulsions, or submicron emulsions, provide kinetic stability and can be created using lower surfactant concentrations (5, 9, 10). SMEDDS, on the other hand, are composed of oils, surfactants, and co-surfactants, which spontaneously disperse to form fine particles upon contact with water, such as in gastrointestinal fluids (11). When hydrophilic surfactants (with a hydrophile-lipophile balance, HLB >12) constitute a significant proportion (≥40% w/w) or are used alongside co-emulsifiers, finer emulsions (around 50 nm in size) can be produced with minimal agitation (12, 13). NE systems require external energy input for stability, whereas SMEDDS undergo spontaneous emulsification, differentiating the two systems in terms of energy and surfactant requirements (14). Recent studies support the effectiveness of these systems. Rodrigues et al. (15) demonstrated that emulsions of *Nectandra grandiflora* essential oil enhanced anesthetic effects and minimized side effects in fish. Kheawfu et al. (7) used Tween 20 to prepare NE and SNEDDS, increasing clove oil permeation through fish skin. Similarly, Khumpirapang et al. (16) designed SNEDDS for *Alpinia galanga* essential oil and found that SNEDDS enhanced the permeation of *A. galanga* oil through fish gills and skin, thereby boosting the anesthetic effect. These studies indicate that NE and SMEDDS can effectively enhance the utilization of essential oils.

*Magnolia denudata*, a decorative tree species native to China (17), has been traditionally used for its analgesic properties, such as relieving headaches (18). Our previous research has shown that *M. denudata* essential oil (MDO) has anesthetic and sedative effects on fish (19, 20). However, research on MDO formulations is limited, and no studies have examined their potential to enhance anesthetic effects in fish. Since gills are the primary organs involved in absorbing anesthetics (21), the surfactants present in NE and SMEDDS may adversely affect the cellular structures of gill cells, particularly the cell membranes (22, 23). With their molecular structure containing hydrophilic heads and hydrophobic tails, surfactants have the potential to disrupt lipid membranes (22, 24). Therefore, the objective of this study is to develop NE and SNEDDS for MDO with high MDO content and defined components to reduce the use of ethanol and surfactants. Moreover, through experimental design optimization, the effects of oil, surfactants, and co-surfactants on the formation of NE and SMEDDS were investigated, further analyzing their stability, the anesthetic and gill physiological effect on fish.

## 2 Materials and methods

### 2.1 Materials

Butanol (98%), ethanol (99.7%), glycerol (99%), isopropanol (99.5%), propylene glycol (99.9%), sorbitan monolaurate (Span 20, 99%, AR), sorbitan monopalmitate (Span 40, 99%, AR), sorbitan monooleate (Span 80, 99%, AR), polysorbate 20 (Tween 20, 99%, AR), and polysorbate 80 (Tween 80, 99%, AR) were all obtained from Shanghai Macklin Biochemical Co., Ltd. MDO (98%) was sourced from Wuhan Kangchun Flavors Co., Ltd.

### 2.2 Miscibility analysis of essential oil and surfactants

This analysis follows methods established in previous research (5). The miscibility of Span 20, Span 80, Tween 20, and Tween 80 with MDO was evaluated by adding 1 g of MDO to 1 g of each surfactant. The mixtures were blended using a vortex mixer for 5 min. After 24 h, if the mixtures remained clear and uniform, they were considered miscible; if they were opaque or showed stratification, they were classified as immiscible.

### 2.3 Preparation of NE

To prepare nanoemulsions (NE), suitable surfactants and their ratios were explored, and energy was applied through ultrasonication. Three surfactants with good miscibility with MDO (Tween 20, Tween 80, and Span 80) were chosen to prepare six NE formulations, consisting of 5% surfactant, 10% or 20% MDO, and 85% or 75% water. The MDO was evenly dispersed in the surfactant-water mixture, stirred at 40–50°C at 1,000 rpm/min, and subjected to ultrasonication at 450 W with a pulse of 5 s on and 5 s off for 6 min using a Scientz-IID ultrasonicator (Xinzhi, China). Formulations that did not separate after 72 h at room temperature were selected for further study. Additional NE formulations with varying MDO (10% or 20%) and surfactant (5–15%) concentrations were prepared and observed under the same conditions. Stable formulations were further characterized, tested for stability, and evaluated for their anesthetic effects.

### 2.4 Preparation of SMEDDS

For the preparation of SMEDDS, the self-emulsification process of MDO was rigorously investigated by analyzing the ratios of water, MDO, surfactants, and co-surfactants. A mixture of surfactant, MDO, and water in a 1:0, 3:5 weight ratio was prepared, resulting in a turbid mixture. Co-surfactants (ethanol, glycerol, isopropanol, propylene glycol, PEG200, and PEG400) were added dropwise until the mixture became clear, and the required amount of co-surfactant was recorded. Pseudo-ternary phase diagrams were constructed with the selected co-solvents using the water titration method at ambient temperature. Initially, mixtures of the surfactant with the selected co-solvent (Smix) were firstly prepared using the weight ratios of 3:1, 2:1, 1:1, 1:2 and 1:3. For each phase diagram, mixtures of MDO and pure surfactant or Smix were prepared at the weight ratios of 0:10, 1:9, 1:8, 1:7, 2:12, 2:10, 2:8, 2:7, 2:6, 3:7, 3:6, 4:6, 5:5, 6:4, 7:3, 8:2, and 9:1. To these mixtures, water was titrated dropwise under gentle mixing using a magnetic stirrer. The component concentrations that yielded transparent mixtures lasting for at least 1 h were used to construct the pseudo-ternary phase diagrams.

### 2.5 Characterization of NE and SMEDDS

Selected formulations, M20-TE5 and SMEDDS, were stored at three different temperatures (4°C, 25°C, and 37°C) for 60 days. The droplet morphology and droplet size were assessed on days 0, 30, and 60 to evaluate stability. For determination of mean droplet size, an aliquot of 1 g of the NE or SMEDDS was diluted with 100 mL of water and gently mixed using a magnetic stirrer at 100 rpm. The mean droplet size of the samples was determined at 25°C by photon correlation spectroscopy (PCS) (Malvern Zetasizer Nano-ZS, Malvern, United Kingdom) at a 173° detector angle. For droplet morphology, 1 g of NE or SMEDDS was diluted with 100 mL water and gently mixed using a magnetic stirrer at 100 rpm. The sample was then placed on a copper grid coated with a carbon polymer film (200 mesh) and dried overnight at 25°C. The grid was loaded into a sample holder of a JEM 2200FS transmission electron microscope (JEOL Co., Japan). Droplet morphology was observed and recorded using a slow-scan CCD camera (Gatan USC1000, Gatan Inc., Pleasanton, CA, United States).

### 2.6 Evaluation of the anesthetic effects and physiological effect of NE and SMEDDS on juvenile *Lateolabrax maculatus*

#### 2.6.1 Fish and sample

Juvenile *L. maculatus* (average weight 153.26 ± 14.89 g) were purchased from a farm in Zhuhai, Guangdong, and acclimated in a pool (4 m × 4 m) for 2 weeks. Water conditions were maintained with a 100% daily exchange, dissolved oxygen at 5.9 ± 0.2 mg/L, temperature at 29 ± 0.5°C, and pH at 7.3 ± 0.1. The fish were subjected to natural light cycles, and water quality was monitored daily to ensure safe parameters. The anesthetic effects of a 100 mg/L MDO ethanol solution (MDO diluted 1:9 in 95% ethanol), M20-TE5, and SMEDDS were compared (19). To avoid the influence of other environmental factors, the water quality parameters of the anesthetic solution were kept consistent with those of the aquaculture water. Fish were fasted for 24 h before the experiment, then placed in buckets containing the respective anesthetic solutions, and only one fish is tested at a time to evaluate the anesthetic effect. The time to reach anesthesia stages A1, A3, and recovery were recorded (Table 1). For stage A3, when the fish has completely lost balance, firmly pinched the tail with forceps. If the fish shows no response, it is considered to have reached the anesthesia stage. After recovery, fish were transferred to anesthetic-free aquariums and sampled 6 h later. Fish were euthanized via cranial concussion at the end of each experiment according to the protocol approved by the Institutional Animal Care and Use Committee of South China Sea Fisheries Research Institute (2021XT06). Gill samples were collected and divided into two groups: small branchial mass of the first pair of branchial arches on the left side for glutathione (GSH) and malondialdehyde (MDA) assay frozen at −80°C and the small branchial mass of the second pair of branchial arches on the left side for morphological examination fixed in 10% formaldehyde.

**Table 1:** Behavioral characteristics of *Lateolabrax maculatus* during anesthesia and recovery stages.

Stage	Behavioral characteristics
A1	Deep sedation: equilibrium normal; total loss of reactivity to external stimuli
A2	Swimming ability disrupted and loss of equilibrium but fish respond to pressure on the caudal peduncle
A3	Deep anesthesia: completely loss of reflex activity or failure to respond to strong external stimuli
A4	Medullary collapse: asphyxia; opercular movements cease
Recovery	Complete recovery of equilibrium; ability to remain upright and normal swimming behavior

#### 2.6.2 GSH and MDA assay

MDA and GSH have been commonly used as effective biomarkers for lipid oxidative stress. The levels of MDA and GSH were determined by using commercially available kits (MDA item: A003-1-2, GSH item: A006-2-1, Nanjing Jiancheng, China) according to the manufacturer’s recommended protocol. Gill tissues were homogenized in 9 volumes (w/v) of 4°C sterile physiological saline and centrifuged at 6,000 × g at 4°C for 20 min. The total protein was determined using the bicinchoninic acid (BCA) assay, where proteins reduce Cu^2+^ to Cu^+^ under alkaline conditions. BCA chelates with Cu^+^ as a chromogenic agent, producing a blue-purple color with an absorption peak at 562 nm. The absorbance at 562 nm is then used to calculate the protein concentration in the sample. Glutathione (GSH) concentration was measured by colorimetry, GSH reacted with 5,5′-dithiobis (2-nitrobenzoic acid) to produce a yellow compound, which can be quantified by colorimetry at 405 nm to determine the concentration of GSH. Malondialdehyde (MDA) concentration was assessed using the thiobarbituric acid assay. Gill supernatant with thiobarbituric acid working solution was incubated at 95°C for 60 min to produce a red compound. The absorbance is then measured at a wavelength of 532 nm.

#### 2.6.3 Histopathology

To further investigate the effects of MDO and eugenol on *L. maculatus*, histological analyses of the gills were conducted 6 h after anesthesia. Gill samples fixed in 10% formaldehyde were washed in 4°C physiological saline, gradually dehydrated with ethanol (70 to 100%), made transparent with xylene, embedded in paraffin, and sectioned into 5–6 μm slices for H&E staining. An optical microscope was used for imaging, and Image Pro Plus 6.0 software was used for image analysis. Five microscope fields of each filament in 5 random gill filaments from each sample and 5 samples for each fish were analyzed. The histopathological analyzes evaluated the distribution of lesions in the analyzed organ and the severity of the alterations according to Bernet et al. (25) The lesions were classified in scores (Sc) and an importance factor (Fi), according to de Castro Sachi et al. (26), the Sc was classified as 0—absence (absence of lesions or lesions on up to 10% of the total analyzed tissue); 1—low frequency (occurrence of lesions from 11 to 25% of the total analyzed tissue); 2—moderate frequency (occurrence of lesions on 26–50% of the analyzed tissue); 3—frequent (occurrence of lesions from 51 to 75% of analyzed tissue); 4—high frequency (occurrence of lesions on 76–100% of the analyzed tissue). The Fi indicates how such an alteration would affect the function of the organ and the possibility of the fish to survive and received the following values: (1) lesions that are easily reversible and of minimal pathological importance; (2) reversible lesions when the stressor is neutralized and moderate pathological importance; (3) lesions that are generally irreversible and of extreme pathological importance. Considering the Sc and Fi for each change, it was calculated the index of each lesions (I_Lesion_), as I_Lesion_ = Fi × Sc and the lesion organ index (I_LOrg_) as I_LOrg_ = ΣI_Lesion_.

### 2.7 Statistical analyses

Anesthetic effects and GSH/MDA assay data are presented as the mean ± standard deviation. Statistical analyses were conducted using SPSS 22.0 software. Data normality and homogeneity of variances were tested and confirmed using the Shapiro–Wilk and Levene tests, respectively. A single-factor analysis of variance was performed, and the Tukey’s HSD multiple comparison method was used to analyze differences. The non-parametric test Kruskal–Wallis followed by Dunn’s post-test was applied to histopathological data to verify differences between treatments and where the difference occurred. A *p*-value <0.05 was considered statistically significant.

## 3 Results

### 3.1 Miscibility analysis of essential oil and surfactants

The mixtures of MDO with Tween 20, Tween 80, and Span 80 at a 1:1 mass ratio was homogeneous and transparent. In contrast, the system comprising MDO and Span 20 exhibited phase separation.

### 3.2 Preparation of NE

Nanoemulsions containing 10 and 20% MDO and 5% surfactant (Tween 20, Tween 80, Span 80) were prepared via ultrasonication. Formulations using Tween 20 and Tween 80 resulted in homogeneous emulsions, whereas those with Span 80 showed oil phase coagulation and layering as indicated in Table 2. Consequently, Tween 20 and Tween 80 were selected for further investigation, including systems with 10 and 20% MDO and 5, 10, and 15% of Tween 20 or Tween 80. Notations used are M for MDO, TT for Tween 20, and TE for Tween 80, with numbers indicating the percentage. Initially, all freshly prepared formulations were homogeneous milky liquids without any instability. However, after 72 h at room temperature, phase separation was observed in the M10-TT15 and all M20-TT systems; the M10-TE15 and M20-TE15 systems also showed layering, as detailed in Table 3. The systems without layering (M10-TT5, M10-TT10, M10-TE5, M10-TE10, M20-TE5, and M10-TE10) were further studied for stability, morphological characterization, and anesthetic effects.

**Table 2:** Change in stability of 5 surfactant-10–20% essence oil mixture after ultrasonic treatment.

Surfactant	Time	10% MDO	20% MDO
Tween 20	72 h after ultrasonic treatment	Uniformity	Uniformity
Tween 80	72 h after ultrasonic treatment	Uniformity	Uniformity
Span 80	72 h after ultrasonic treatment	Heterodisperse	Heterodisperse

**Table 3:** Change in stability of 5–15% surfactant-10–20% essence oil mixture after ultrasonic treatment.

MDO ratio	Time	5% Tween 20	10% Tween 20	15% Tween 20	5% Tween 80	10% Tween 80	15% Tween 80
10%	72 h after ultrasonic treatment	Heterodisperse	Uniformity	Heterodisperse	Uniformity	Uniformity	Uniformity
20%	72 h after ultrasonic treatment	Heterodisperse	Heterodisperse	Heterodisperse	Uniformity	Uniformity	Uniformity

### 3.3 Preparation of SMEDDS

Based on the outcomes from the nanoemulsion preparations, Tween 80 was selected as the surfactant for the SMEDDS. A transparent system was obtained by incorporating 10% ethanol, 15% isopropanol, or 35% propylene glycol into a mixture of Tween 80, MDO, and water at a weight ratio of 1:0.3:5. Under identical conditions, other co-solvents such as butanol, PEG200, PEG400, and glycerol did not facilitate the formation of microemulsions at concentrations below 35%. Consequently, ethanol was chosen as the co-surfactant for SMEDDS.

**Figure 1:**
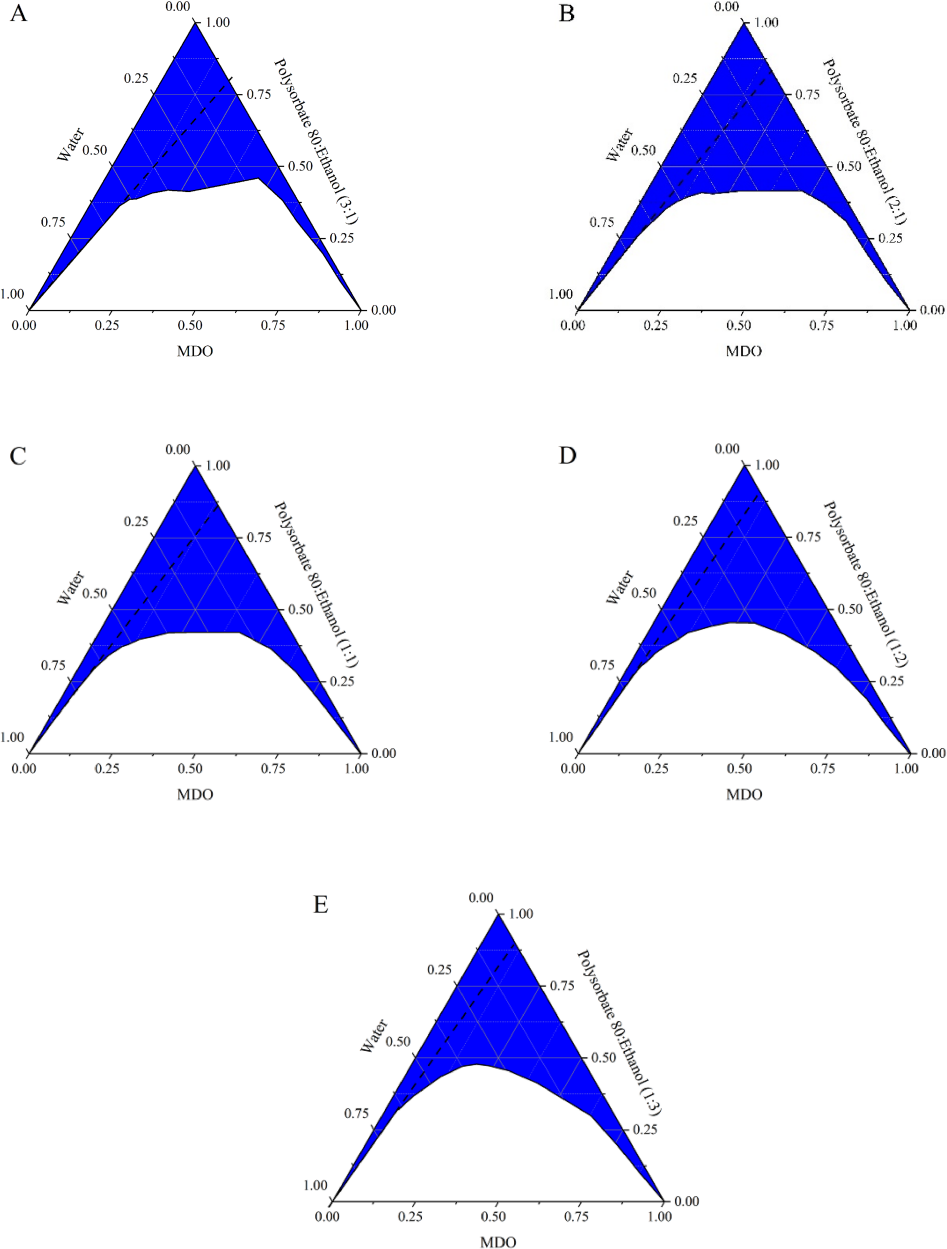
Pseudo-ternary phase diagrams of the systems composed of MDO, water, and different Km ratio mixed surfactant. (A) Tween 80: ethanol (3:1); (B) Tween 80: ethanol (2:1); (C) Tween 80: ethanol (1:1); (D) Tween 80: ethanol (1:2); (E) Tween 80: ethanol (1:3).

The trend in the microemulsion region first expanded and then contracted with decreasing Km values, as depicted in Figure 1, while the area available for dilution without lines diminished. Based on the microemulsion region, the dilution area without lines, and the usage of anhydrous ethanol, formulations with 10, 20, and 30% MDO and a Km of 2:1 (labeled MS-1, MS-2, and MS-3) were identified for further studies on stability, morphological characterization, and anesthetic effects.

### 3.4 Stability testing

After 7 days at both 4°C and 37°C, phase separation was observed in M20-TE10, M10-TE5, M10-TE10, M20-TT5, and M20-TT10. Only M20-TE5 demonstrated no signs of instability throughout the experimental period. After storage for 60 days, there was an increase in the particle size of M20-TE5, and a general increase in the particle size of all SMEDDSs at 37°C, as detailed in Table 4.

**Table 4:** Droplet size of NE and SMEDDS formulations stored at 4, 30, and 37°C for a period of 60 days (nm).

Formulations	0 days	30 days			60 days		
		4°C	25°C	37°C	4°C	25°C	37°C
M20-TE5	231.16 ± 2.17	292.00 ± 4.45	375.04 ± 11.45	466.40 ± 14.59	353.06 ± 6.63	440.75 ± 9.54	650.46 ± 26.11
MS-1	9.13 ± 0.13	9.18 ± 0.21	10.40 ± 0.17	11.08 ± 0.75	9.44 ± 0.37	10.51 ± 0.26	10.73 ± 0.61
MS-2	92.83 ± 3.09	95.00 ± 1.48	102.07 ± 1.80	115.98 ± 4.27	97.23 ± 1.24	105.47 ± 1.23	136.51 ± 4.38
MS-3	281.06 ± 4.6	285.51 ± 2.32	302.15 ± 6.09	320.50 ± 7.26	285.07 ± 0.4.37	306.56 ± 5.67	351.26 ± 13.14

### 3.5 TEM morphological observation

**Figure 2:**
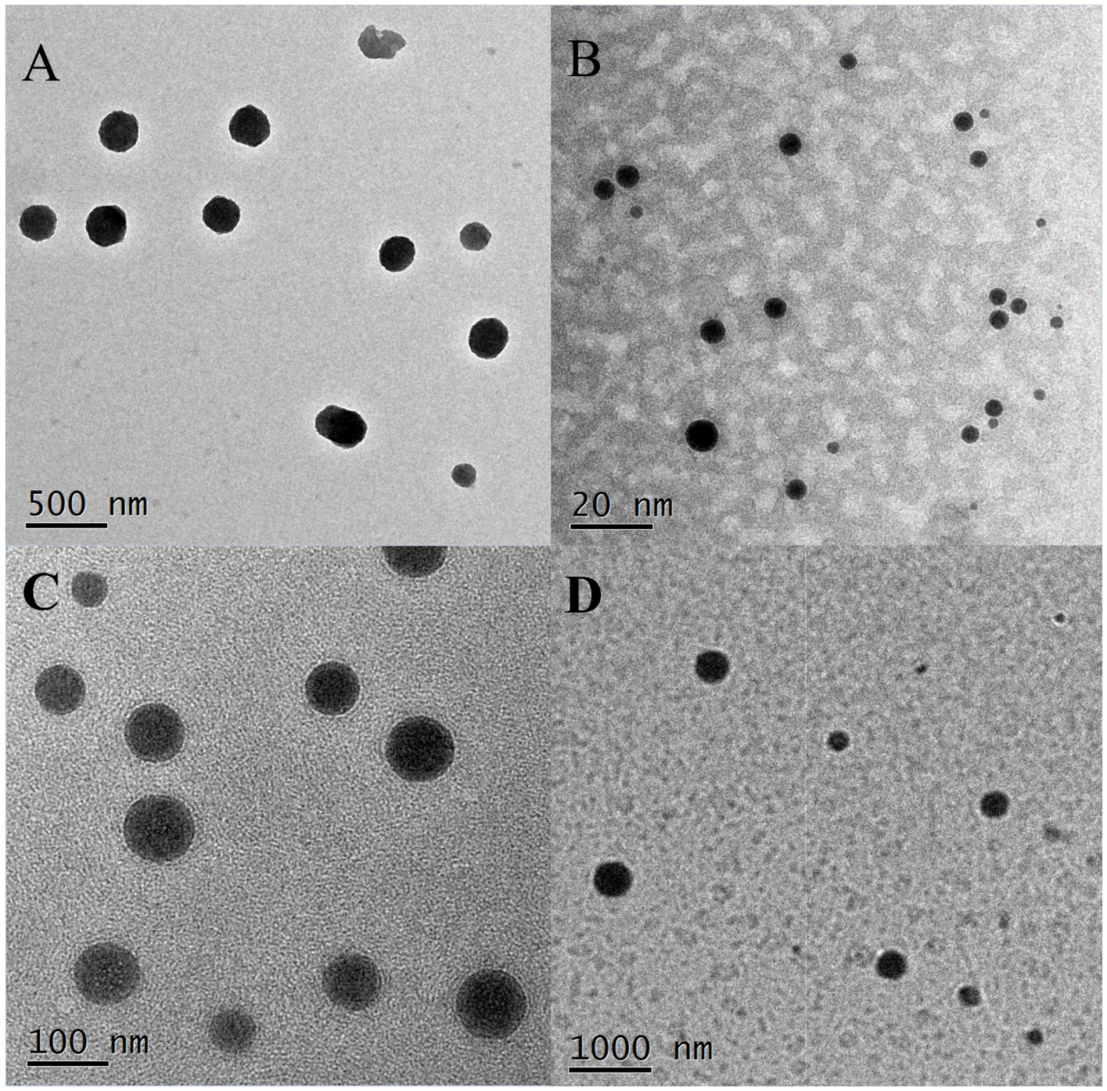
Morphology of NE and SMEDDS. (A) M20-TE5 (500 nm, 20,000×); (B) MS-1 (20 nm, 200,000×); (C) MS-2 (100 nm, 50,000×); (D): MS-3 (1,000 nm, 10,000×).

TEM images revealed that the droplets of M20-TE5 and SMEDDS were spherical, with a smooth appearance and size consistent with particle size analysis, as shown in Figure 2.

### 3.6 Evaluation of the anesthetic effects and physiological effect of NE and SMEDDS on juvenile *Lateolabrax maculatus*

#### 3.6.1 Evaluation of the anesthetic effects of NE and SMEDDS on juvenile *Lateolabrax maculatus*

**Figure 3:**
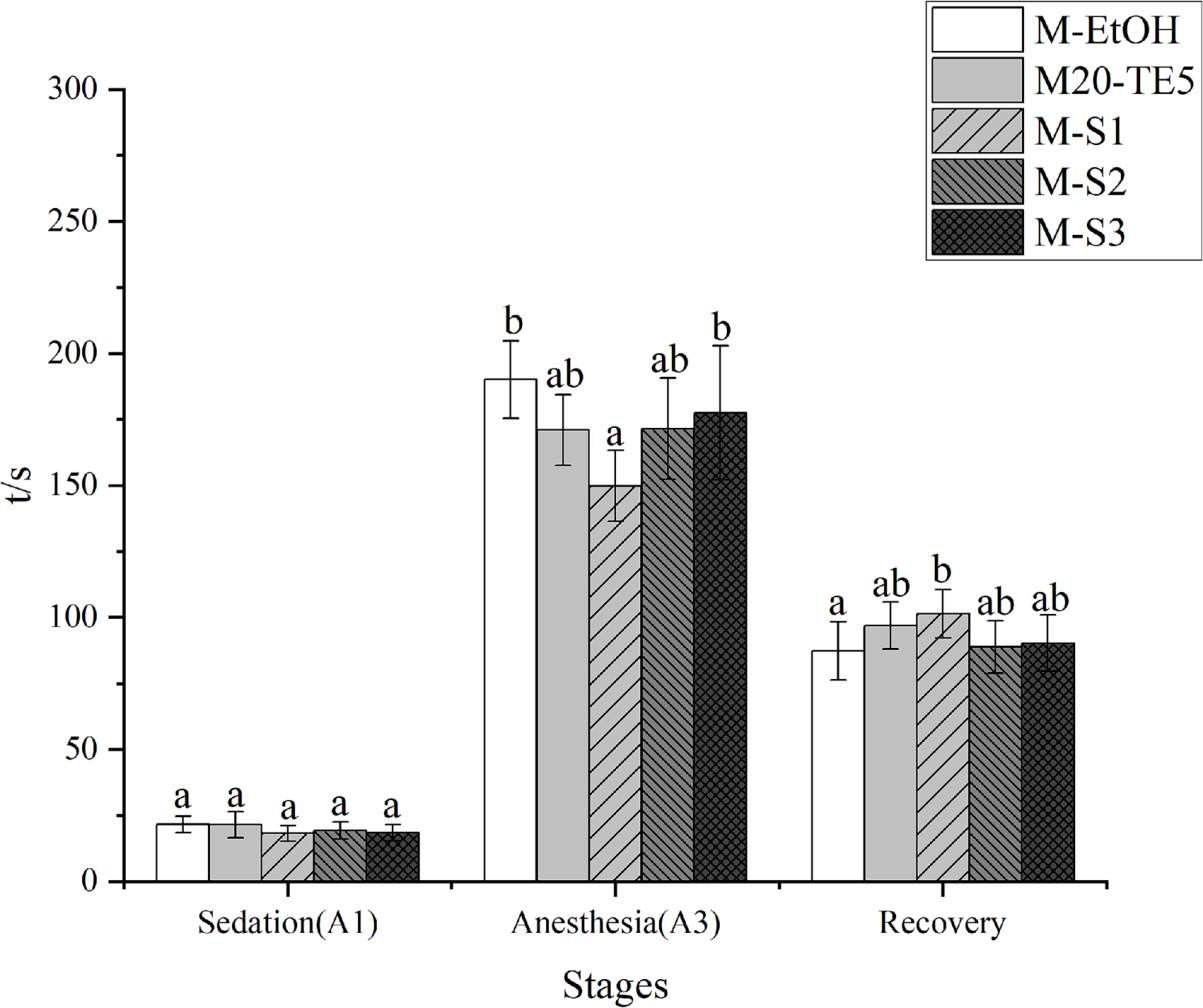
Anesthesia efficiency of NE and SMEDDS on *L. mulucaltus*. Different letters above the bars indicate significant difference (*n* = 10; *p* < 0.05).

As depicted in Figure 3, the time to reach sedation was reduced for the M20-TE5 and SMEDDS groups compared to the ethanol group, though the difference was not statistically significant (*p* > 0.05). However, both NE and SMEDDS formulations decreased the time required to induce anesthesia, with the M-S1 group significantly lower than that of ethanol (*p* < 0.05). Furthermore, the recovery time for the M-S1 group was significantly longer compared to the ethanol group (*p* < 0.05).

#### 3.6.2 GSH and MDA assay

**Figure 4:**
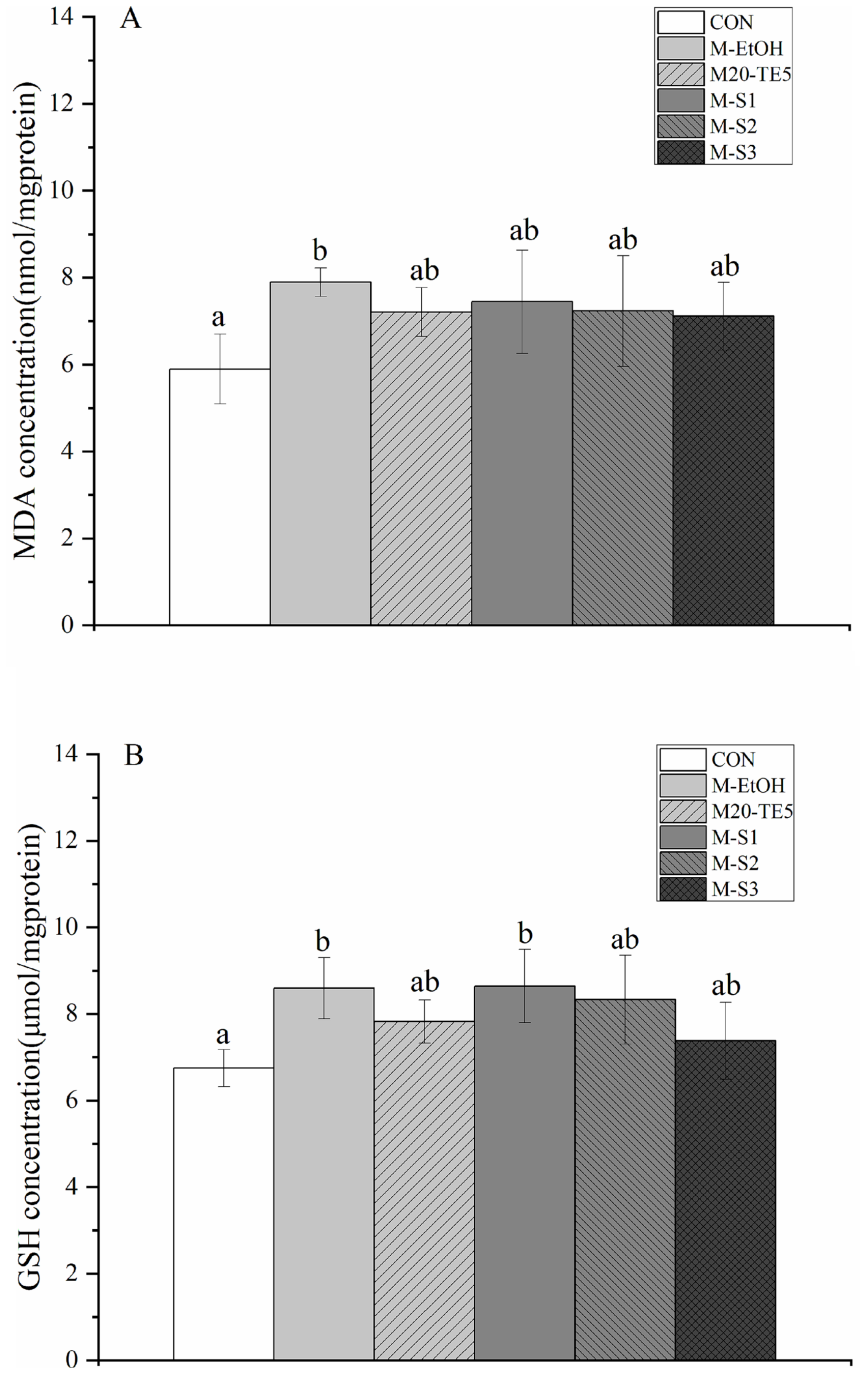
Change of GSH and MDA concentration after NE and SMEDDS anesthesia on *L. mulucaltus* (*n* = 4; *p* < 0.05). (A) MDA; (B) GSH.

In all anesthetized fish, the M-EtOH group showing significantly higher levels than the control group (*p* < 0.05). However, there were no significant differences in MDA levels between the M20-TE5, SMEDDS, M-EtOH and control groups (*p* > 0.05, Figure 4A).

Additionally, the M-EtOH and M-S1 groups exhibited GSH levels significantly higher than those in the control group (*p* < 0.05). The M20-TE5, M-S2 and M-S3 group did not show a significant difference in GSH levels compared to the control group (*p* > 0.05, Figure 4B).

#### 3.6.3 Histopathology

**Figure 5:**
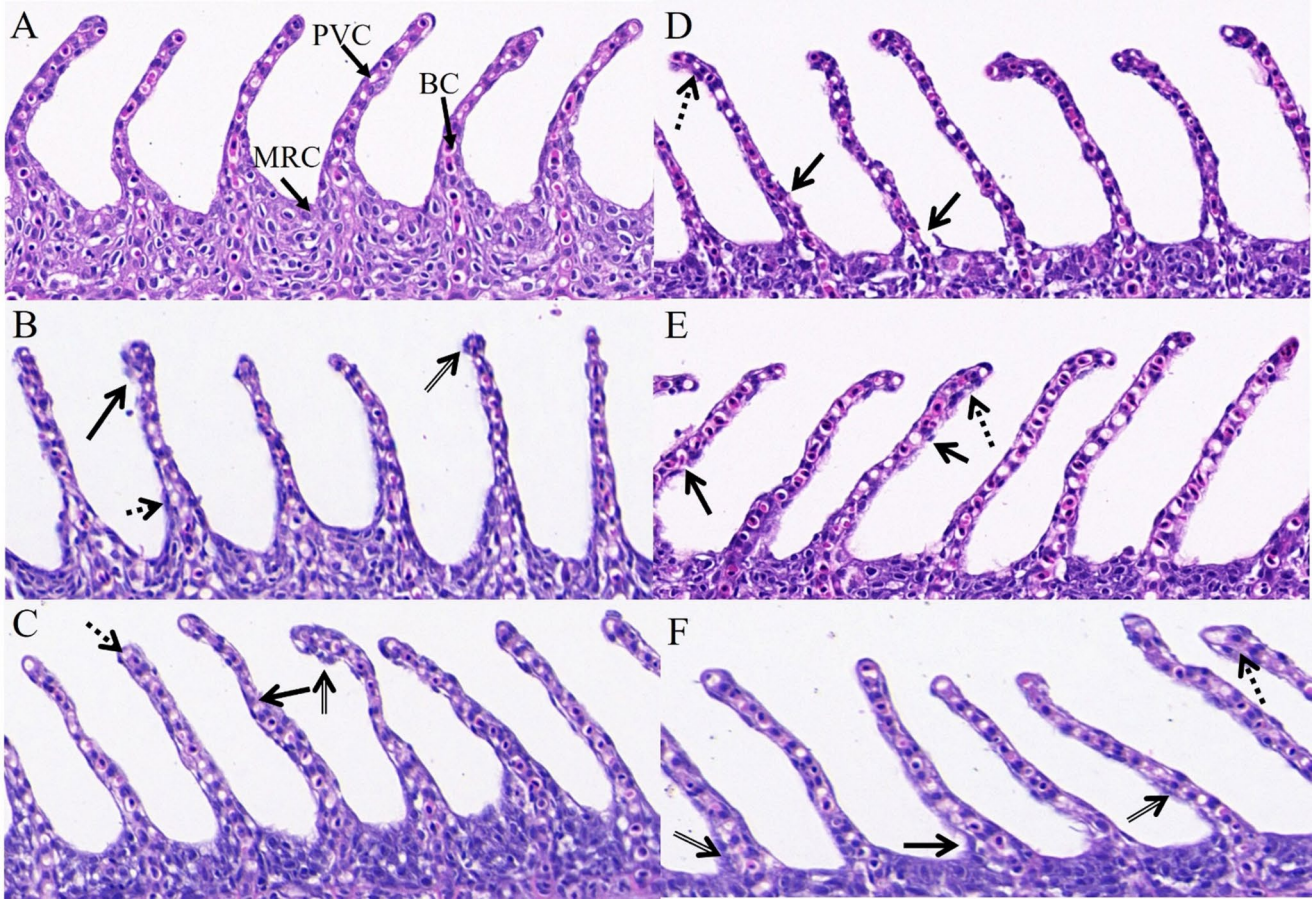
Observation on gills in different groups. PVC, pavement cells; BV, blood vessel; MRC, erythrocytes. Scale bar: 50 μm; (10 × 40 times); solid arrow (rupture of lamellar epithelium); dotted arrow (lamellar epithelium detachment and edema); double solid arrow (lamellar epithelium hyperplasia). (A) CON; (B) M-EtOH; (C) M20-TE5; (D) M-S1; (E) M-S2; (F) M-S3.

In the control group, the fish gills were intact, extending laterally, with flat cells arranged regularly and red blood cells distributed evenly. There were no signs of hyperplasia or edema, indicating a normal physiological state. In contrast, the M-EtOH, NE and SMEDDS exhibited epithelial cell hyperplasia, swelling, and rupture; the arrangement of gill filaments remained relatively orderly (Figure 5). In the evaluation of the gill lesion index, the ethanol co-solvent group showed significantly higher lamellar epithelium hyperplasia and rupture of the lamellar epithelium compared to the control group (*p* < 0.05). However, no significant changes were observed in the NE and SMEDDS groups (*p* > 0.05, Table 5).

**Table 5:** Gill alteration index (I_Lesion_) in control groups and exposed groups to M-EtOH, M20-TE5, M-S1, M-S2, and M-S3.

Alterations	Gill lesion index (I_lesion_)						
	Fi	Control	M-EtOH	M20-TE5	M-S1	M-S2	M-S3
Aneurysm (apical marginal channel and lamellar)	1	1.0 ± 0.0	1.2 ± 0.2	1.1 ± 0.1	1.1 ± 0.2	1.2 ± 0.3	1.1 ± 0.1
Congestion/hyperemia/hemorrhage	1	1.0 ± 0.0	1.3 ± 0.3	1.2 ± 0.3	1.2 ± 0.1	1.1 ± 0.1	1.0 ± 0.0
Cellular atrophy	2	2.1 ± 0.1	2.4 ± 0.3	2.1 ± 0.1	2.4 ± 0.3	2.3 ± 0.1	2.1 ± 0.2
Filament epithelium	1	1.0 ± 0.2	1.1 ± 0.1	1.2 ± 0.2	1.1 ± 0.1	1.0 ± 0.0	1.4 ± 0.2
Pillar cells (constriction/rupture/marginal channel dilatation)	1	1.0 ± 0.1	1.2 ± 0.2	1.2 ± 0.2	1.1 ± 0.1	1.3 ± 0.2	1.1 ± 0.1
Lamellar epithelium detachment and edema	1	1.0 ± 0.0	1.4 ± 0.4	1.2 ± 0.1	1.0 ± 0.1	1.2 ± 0.1	1.1 ± 0.1
Partial fusion of lamellae	1	1.0 ± 0.0	1.0 ± 0.0	1.0 ± 0.0	1.0 ± 0.0	1.0 ± 0.0	1.1 ± 0.1
Total fusion of lamellae	2	2.0 ± 0.0	2.0 ± 0.0	2.0 ± 0.0	2.0 ± 0.0	2.0 ± 0.0	2.0 ± 0.0
Lamellar epithelium hyperplasia	1	1.0 ± 0.0	1.8 ± 0.2^a^	1.3 ± 0.2	1.4 ± 0.4	1.3 ± 0.3	1.3 ± 0.4
Lamellar epithelium hypertrophy	2	2.0 ± 0.0	2.3 ± 0.3	2.2 ± 0.3	2.2 ± 0.3	2.1 ± 0.2	2.2 ± 0.3
Cellular necrosis (focal/total)	3	3.0 ± 0.0	3.0 ± 0.0	3.0 ± 0.0	3.0 ± 0.0	3.0 ± 0.0	3.0 ± 0.0
Rupture of lamellar epithelium	2	2.0 ± 0.1	2.8 ± 0.3^a^	2.1 ± 0.2	2.3 ± 0.2	2.4 ± 0.4	2.2 ± 0.1

Fi, importance factor.

a Indicates significant differences from the respective control group (*p* < 0.05).

## 4 Discussion

Surfactants, with their hydrophilic and lipophilic properties, enhance the solubility of essential oils in water and help disperse them into nanoparticles (9). Currently, a multitude of surfactants are utilized for the preparation of essential oil nanoemulsions, including Tween, Span, chitosan, and zein protein (14, 27). Non-ionic surfactants are generally considered to have the lowest toxicity, making the Tween and Span series popular choices for this experiment with MDO. However, during compatibility testing and ultrasonic emulsification, Span surfactants exhibited instability, likely due to their lack of polyoxyethylene groups, which are essential for binding to small molecules like linalool in MDO (28). Additionally, emulsions containing Tween 80 showed greater stability after ultrasonic emulsification compared to those with Tween 20, aligning with findings from Pavoni et al. (14), who noted that Tween 80 stabilizes essential oil emulsions more effectively and with less toxicity. Consequently, Tween 80 was selected as the primary surfactant for SMEDDS in this study to reduce the need for co-surfactants and minimize negative effects on fish.

Co-surfactants play a crucial role in adjusting the surface activity and hydrophilic–lipophilic balance of surfactants, aiding micelle formation and modulating the polarity of water and oil (9, 13). Ethanol was chosen as the co-surfactant for this study due to its effectiveness in stabilizing emulsions. As the Km value decreased, the microemulsion region initially expanded and then contracted, with the largest areas observed at Km values of 2:1 and 1:1. These ratios allowed for a balance between anhydrous ethanol usage and the microemulsion area, making Km values of 2:1 and MDO concentrations of 10, 20, and 30% ideal for further study.

Transmission electron microscopy revealed that the droplets in both NE and SMEDDS were spherical, with NE droplet sizes averaging around 200 nm. In contrast, SMEDDS droplets ranged from 10 nm to approximately 300 nm as the essential oil concentration increased, consistent with the behavior of self-emulsifying systems. Over 60 days of storage at various temperatures, NE particle size significantly increased, potentially due to the destabilization of the ethoxylation chains in Tween 80, which reduced its binding capacity with small molecules in the essential oil (29). However, SMEDDS formulations exhibited less particle size variation, likely because the higher proportions of co-surfactants and surfactants improved the essential oil’s solubility in aqueous solutions and reduced surface tension (5).

The efficacy of anesthetics depends on factors such as species, fish size, and environmental temperature, with optimal concentrations typically inducing anesthesia within 3 min (1, 3, 30). In this study, MDO at 100 mg/L in ethanol served as the control, while NE and SMEDDS formulations were tested in induction experiments on fish. Both NE and SMEDDS groups demonstrated shorter induction times and longer recovery periods. Since gills and skin are the primary organs through which fish absorb anesthetics, surfactants, by altering membrane permeability, can enhance drug penetration (31). Previous studies have also shown that smaller particle sizes facilitate drug absorption (16). The surfactants and co-surfactants in NE and SMEDDS decreased the surface tension of MDO, reduced particle size, and increased its surface area, thus enhancing the permeability of gills and skin to MDO and promoting absorption, ultimately boosting the anesthetic effect.

Further evaluation of the physiological impact of NE and SMEDDS on gill tissues is essential. The GSH is an important non-enzymatic antioxidant in cells, which can neutralize free radicals or oxidants (32). The MDA is the most important marker of lipid peroxidation (33). Both GSH and MDA are commonly used biomarkers for oxidative stress (20). In this study, elevated levels of MDA and GSH in the ethanol co-solvent group were consistent with previous research. However, lower MDA and GSH levels were observed between the NE, SMEDDS, and control groups compared to ethanol co-solvent group, indicating that gill tissue exposed to NE and SMDESS may be received less oxidative stress. Morphological changes were observed in the gill tissues of all anesthetized fish. However, the changes observed in the NE and SMEDDS groups were mild and did not affect physiological function (25, 36). Some studies have found that using other types of surfactants for the nanonization of essential oils can effectively reduce negative effects on fish. Tween 20 and 80, when used as co-solvents with *Lippia alba* essential oil, neither increased nor decreased cytotoxicity to blood cells but did enhance the anesthetic effect (34). Rodrigues et al. (15) observed that essential oil anesthetics encapsulated with Tween 80 and subjected to long exposure times had high survival rates in nano-oil formats. Shah et al. (35) suggested that short exposure times to NE with fast anesthetic induction are beneficial in reducing stress. Therefore, we hypothesize two reasons for the lower stress-induced damage to the gills in the NE and SMEDDS groups. On the one hand, the lower concentration of surfactants in NE and SMEDDS likely caused less stress to the gills. On the other hand, the shorter anesthesia induction time reduced the gills’ exposure to the anesthetic agents. In conclusion, NE and SMEDDS represent a reliable and safe delivery system for essential oil anesthetics. They effectively mediate anesthetic effects without adding additional stress or toxicity to aquatic organisms, making them suitable for applications requiring minimal physiological disruption.

## 5 Conclusion

Our study successfully developed two types of MDO-based nanoemulsions, NE and SMEDDS, and assessed their physicochemical properties. The anesthesia experiments demonstrated that both NE and SMEDDS significantly enhanced the anesthetic effects of MDO. Moreover, further investigations into their physiological impact on fish gills confirmed that these nanoemulsions are safe anesthetic formulations. These findings are particularly important as they highlight not only the efficacy of NE and SMEDDS in delivering anesthetic agents but also their properties that mitigate toxicity, especially concerning vital respiratory organs like the gills. Such attributes are critical for applications in veterinary medicine, particularly in aquaculture and research environments, where the welfare and survival of aquatic species are top priorities.

## Data Availability

The original contributions presented in the study are included in the article/supplementary material, further inquiries can be directed to the corresponding authors.
